# Harnessing HfO_2_ Nanoparticles for Wearable Tumor Monitoring and Sonodynamic Therapy in Advancing Cancer Care

**DOI:** 10.1021/acsnano.3c11346

**Published:** 2024-01-10

**Authors:** Putry Yosefa Siboro, Amit Kumar Sharma, Pei-Jhun Lai, Jayachandran Jayakumar, Fwu-Long Mi, Hsin-Lung Chen, Yen Chang, Hsing-Wen Sung

**Affiliations:** † Department of Chemical Engineering, National Tsing Hua University, Hsinchu 30013, Taiwan (ROC); ‡ Department of Biochemistry and Molecular Cell Biology, School of Medicine, College of Medicine, Taipei Medical University, Taipei 23142, Taiwan (ROC); § Taipei Tzu Chi Hospital, Buddhist Tzu Chi Medical Foundation and School of Medicine, Tzu Chi University, Hualien 97004, Taiwan (ROC)

**Keywords:** tumor progression monitoring, hafnium oxide nanoparticle, wearable strain sensor, cancer care, telemedicine

## Abstract

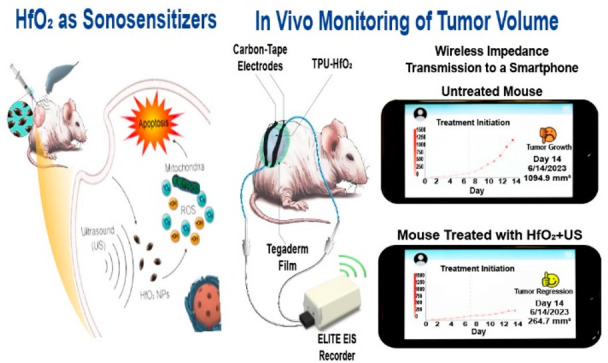

Addressing the critical requirement for real-time monitoring of tumor progression in cancer care, this study introduces an innovative wearable platform. This platform employs a thermoplastic polyurethane (TPU) film embedded with hafnium oxide nanoparticles (HfO_2_ NPs) to facilitate dynamic tracking of tumor growth and regression in real time. Significantly, the synthesized HfO_2_ NPs exhibit promising characteristics as effective sonosensitizers, holding the potential to efficiently eliminate cancer cells through ultrasound irradiation. The TPU-HfO_2_ film, acting as a dielectric elastomer (DE) strain sensor, undergoes proportional deformation in response to changes in the tumor volume, thereby influencing its electrical impedance. This distinctive behavior empowers the DE strain sensor to continuously and accurately monitor alterations in tumor volume, determining the optimal timing for initiating HfO_2_ NP treatment, optimizing dosages, and assessing treatment effectiveness. Seamless integration with a wireless system allows instant transmission of detected electrical impedances to a smartphone for real-time data processing and visualization, enabling immediate patient monitoring and timely intervention by remote medical staff. By combining the dynamic tumor monitoring capabilities of the TPU-HfO_2_ film with the sonosensitizer potential of HfO_2_ NPs, this approach propels cancer care into the realm of telemedicine, representing a significant advancement in patient treatment.

The absence of techniques to monitor the progression of tumors in real-time presents a significant challenge in cancer care. The volume of a tumor can be a valuable indicator of its development and progression. Identifying a reliable method to continuously detect changes in tumor volume could lead to a better understanding of tumor development. This, in turn, would empower clinicians to take prompt action during the critical stages of cancer treatment and result in improved patient care.

In clinical practice, measuring tumor volumes is commonly done using computed tomography (CT) scanners or mechanical instruments such as calipers.^1,2^ However, standard *in vivo* CT scans typically assess only two dimensions, width and length, despite tumors being three-dimensional (3D) entities, encompassing width, length, and height. While recent advancements include the use of multidetector CT scans for reconstructing 3D tumor images, their extensive clinical use remains limited due to their size and cost.^3^ Conversely, although calipers can measure all three diameters, they may produce measurement errors of up to 20% due to variations in tumor shape.^4^ Therefore, accurately detecting changes in tumor volume to track its temporal growth and regression has become a challenging task.

Hafnium oxide (HfO_2_) is a dielectric material that has a high dielectric constant (κ).^5^ High-κ dielectric materials are widely used in various electronic devices, including capacitors.^6^ Its biocompatibility, stability, and low toxicity have recently garnered attention in the medical field, making it an attractive material for various applications.^7^ One such application is using HfO_2_ as a sensing material to detect gases and other analytes.^8^ This is achieved by monitoring the adsorption or desorption of analyte molecules on the HfO_2_ surface, which alters its capacitance and results in changes in the electrical impedance. Another exciting application of HfO_2_ involves NBTXR3, the first-in-class HfO_2_-based nanoparticles (NPs) designed for direct intratumoral injection as radiosensitizers to enhance the effects of radiotherapy.^9^ Hf demonstrates an exceptional impact in significantly improving the efficacy of radiotherapy within targeted tumor regions.^10^ Furthermore, it contributes to an increased generation of cytotoxic hydroxyl radicals (•OH) and other reactive oxygen species (ROS) within the tumor microenvironment.^11^ This heightened ROS production plays a crucial role in facilitating the precise and comprehensive elimination of tumor cells.

Sonodynamic therapy (SDT) offers a promising avenue for cancer treatment, employing ultrasound (US) in conjunction with sonosensitizer agents to selectively target tumor tissues while minimizing harm to healthy counterparts. In the course of SDT, sonosensitizers, such as titanium oxide (TiO_2_) NPs,^12^ react to US waves within the tumor, catalyzing interactions with adjacent oxygen and water molecules. This interplay gives rise to potent ROS that effectively eliminates cancer cells. Much like their TiO_2_ counterparts, HfO_2_ NPs are part of the metal oxide category and exhibit comparable properties,^13^ indicating their potential as inorganic sonosensitizers for SDT—able to generate ROS when exposed to US waves. Given that HfO_2_ NPs have already secured Food and Drug Administration (FDA) approval for intratumoral administration, they emerge as prime candidates for serving as sonosensitizers in the ongoing exploration of SDT within this study.

This work proposes a wearable platform for on-body monitoring of tumor growth and regression using a thermoplastic polyurethane (TPU) film that incorporates HfO_2_ NPs (forming a TPU-HfO_2_ composite film) as a dielectric elastomer (DE) sensor (Figure 1). The primary objective of this platform is to improve cancer care by enabling real-time monitoring of tumor volume changes both prior to and following the therapeutic application of HfO_2_ NPs in SDT. This capability is pivotal for precise treatment initiation and dosing for smaller tumor volumes as well as for evaluating treatment effectiveness at larger volumes.

**Figure 1:**
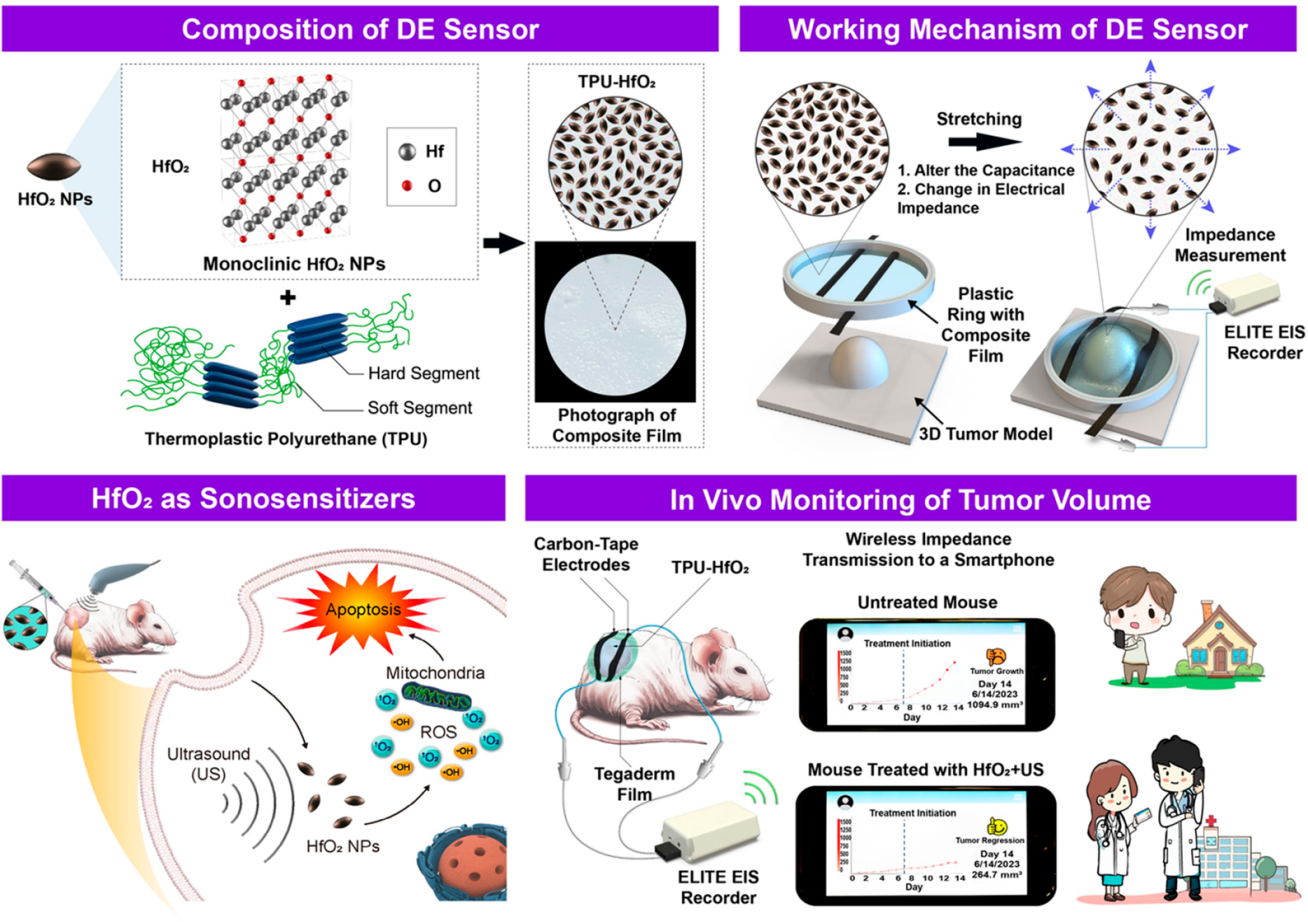
Preparation and working mechanism of DE stain sensor composed of TPU and HfO_2_ NPs that could undergo deformation in multidirections as a response to tumor progression, leading to alterations in its electrical impedance. The DE strain sensor is integrated into a smartphone, enabling convenient tracking of the tumor size. Additionally, it remotely notifies healthcare professionals, facilitating the intervention through HfO_2_ NPs-based sonodynamic therapy.

TPU, a segmented-block copolymer comprising soft and hard segments arranged alternately, combines the processability of thermoplastics with the elasticity of rubber.^14^ It has found widespread use in biomedical applications.^15^ TPU films are an excellent option for on-body applications due to their biocompatibility and exceptional mechanical properties, such as high elasticity and durability. Additionally, they are highly flexible and stretchable, making them well-suited for use in wearable and pliable strain sensors that collect and analyze real-time data related to various physiological factors.^16^ By incorporation of electronic components, TPU films can help individuals make informed decisions about their health and well-being.

The TPU-HfO_2_ composite film operates as a DE strain sensor, providing continuous monitoring of tumor growth and regression. Fitted with flexible carbon-tape electrodes, this sensor is positioned atop the tumor, engaging with an applied electrical field to enable seamless monitoring. As the tumor volume undergoes changes, the DE sensor experiences a mechanical deformation. This deformation leads to significant adjustments in the spatial distribution and arrangement of the HfO_2_ NPs that are embedded within the TPU film structure. The shifting proximity of these NPs induces changes in their respective electric fields, which impact their dielectric properties.^17^ This dynamic process influences the capacitance of the TPU-HfO_2_ composite film, generating variations in its electrical impedance. Effectively, this interactive process provides immediate insights into the degree of deformation or strain arising from changes in the tumor volume.

Opting for a xenograft tumor model enables the establishment of a versatile framework for continuously monitoring the progression of subcutaneous tumors, including basal cell carcinoma, squamous cell carcinoma, and melanoma. While basic visual observation provides some insights, its accuracy in tracking tumor size changes over time might be limited. Developing a more systematic and objective monitoring method significantly enhances the precision and effectiveness of tracking tumor progression.

The proposed wearable TPU-HfO_2_ composite film (DE sensor) is integrated with a WI-FI-based system to wirelessly collect and transmit electrical impedance data using a smartphone in the patient’s personal environment. The collected impedance data are processed by an app program installed on the smartphone, providing the patient with real-time information about the progression of the tumor. The information can also be transmitted from the patient’s smartphone to remote medical professionals for real-time intervention via the Internet, thus advancing the field of cancer care into the realm of telemedicine. With this innovation, the optimal timing for initiating HfO_2_ NPs treatment for sonodynamic therapy, optimization of the dosage, and evaluation of the treatment’s effectiveness can be determined. The cross-era significance of this method lies in its potential to revolutionize cancer care by making it more accessible and efficient through remote monitoring and intervention.

## Results and Discussion

### Characteristics of HfO_2_ Particles

By carefully controlling the synthesis of HfO_2_ particles and tuning their size and morphology, it is possible to optimize their dielectric properties for specific sensing applications. HfO_2_ particles with a high dielectric constant are particularly desirable, as they can enhance the sensitivity and accuracy of the as-proposed DE strain sensor.

**Figure 2:**
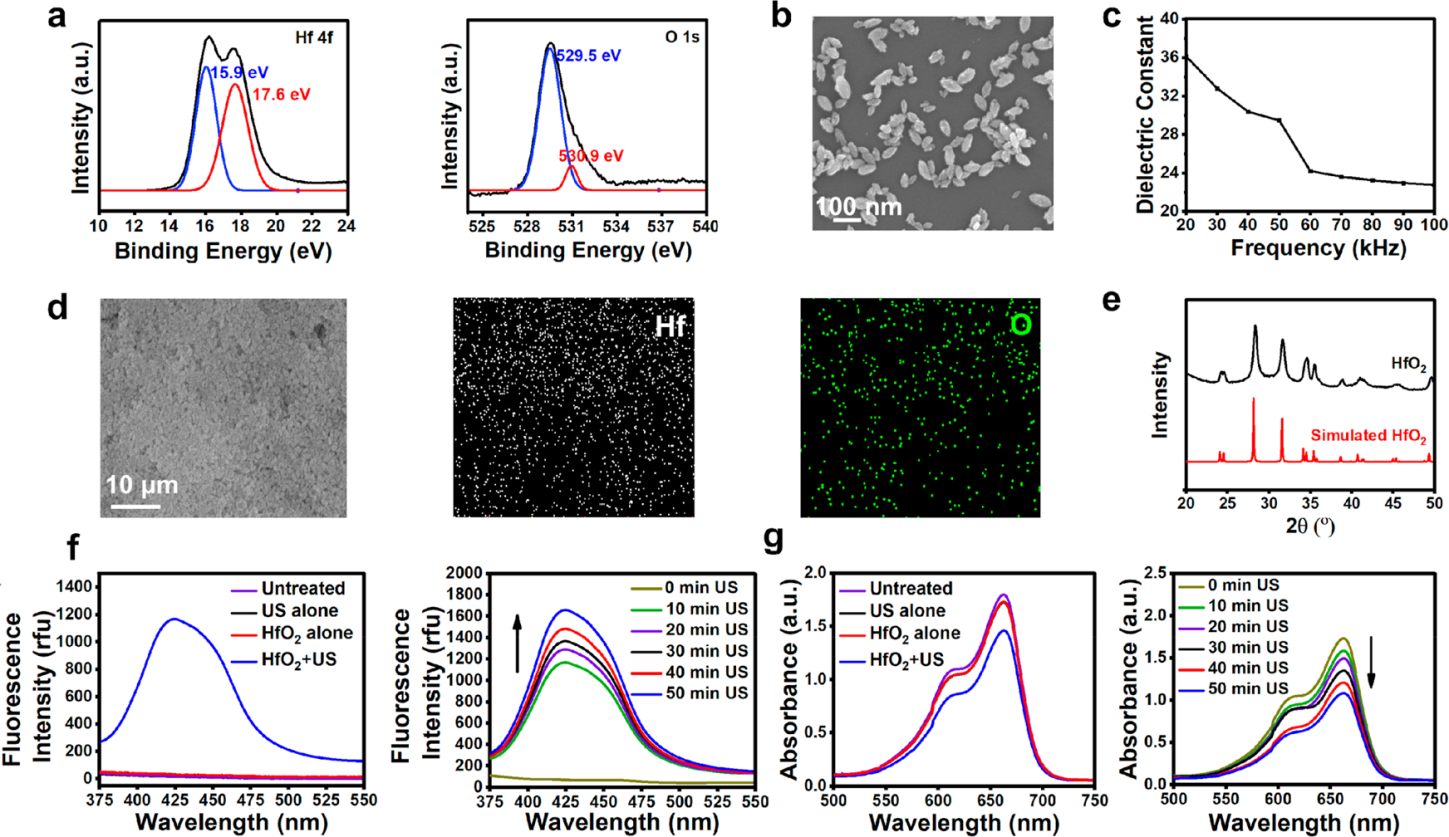
Characteristics of HfO_2_ NPs. (a) Hf 4f XPS spectra deconvoluted into Hf 4f_5/2_ (red) and Hf 4f_7/2_ (blue); and the O 1s XPS spectra deconvoluted into two main peaks at 529.5 (blue) and 530.9 eV (red). (b) SEM micrograph of HfO_2_ NPs. (c) Frequency-dependent dielectric properties of HfO_2_ NPs. (d) EDS elemental mapping images displaying even distribution of Hf and O in HfO_2_ NPs. (e) XRD patterns of HfO_2_ NPs and simulated monoclinic HfO_2_ NPs. (f) Fluorescence spectra of TA in different treatment groups and at various reaction times. (g) Absorption spectra of MB under distinct treatment conditions and varying reaction times.

In this study, HfO_2_ particles were synthesized via a hydrothermal reaction between hafnium chloride (HfCl_4_) and sodium hydroxide (NaOH) in an aqueous solution at 120 °C for 20 h.^18^ The chemical composition and valence states of Hf and O in the as-synthesized HfO_2_ particles were investigated using X-ray photoelectron spectroscopy (XPS). The results demonstrated the presence of Hf–O bonding in the particles (Figure 2a). The Hf 4f spectra showed two distinct peaks at 15.9 and 17.6 eV, corresponding to Hf 4f_7/2_ and Hf 4f_5/2_ peaks of the Hf oxide bond (O–Hf–O), respectively,^5^ which suggests the existence of Hf–O bonds in the HfO_2_ particles. Notably, the absence of low-energy peaks in the data indicates the presence of stoichiometric HfO_2_.^19^ In addition, the O 1s spectra revealed a major peak at 529.5 eV and a shoulder peak at 530.9 eV, consistent with the presence of Hf–O bonding in the material. These findings indicate that the Hf–O bond significantly contributes to the chemical bonding in the HfO_2_ particles.

The HfO_2_ particles exhibited a spindle-like morphology, as shown in Figure 2b through scanning electron microscopy (SEM). Due to their more elongated shape, spindle-like particles may have a higher dielectric constant than their spherical counterparts as they can achieve higher surface area and surface polarization.^20^ The size of the HfO_2_ particles was determined through SEM analysis and analyzed with ImageJ software (Figure S1), revealing an average length of 99.5 ± 12.6 nm and a diameter perpendicular to the long axis of 55.8 ± 13.8 nm (*n* = 50 particles). Additionally, their zeta potential value (Figure S2) was measured using dynamic light scattering (DLS) and found to be −31.3 ± 1.1 mV (*n* = 6 batches). It is worth noting that nanosize particles, regardless of their morphology, typically have a higher dielectric constant than their microsize counterparts due to the quantum confinement effect.^21^

The dielectric constant of a material plays a crucial role in sensing applications that demand high sensitivity and accuracy.^22^ In the study, it was observed that the dielectric constant of the spindle-like HfO_2_ NPs synthesized decreased as the frequency of the applied electric field increased (Figure 2c). This decrease can be attributed to the weakened polarization between the interfaces with the increased frequency, resulting in a decline in the dielectric properties.^23^ Of particular note is the measured value of approximately 23.0 at a frequency of 100 kHz, which serves as a representative value for the dielectric property of the as-synthesized HfO_2_ NPs. Frequencies around 100 kHz are commonly employed for measuring the dielectric constant of inorganic particles.^24^ This choice of frequency enables precise measurements, while also minimizing potential interference from other factors. To confirm the element distribution within the HfO_2_ particles, energy-dispersive X-ray spectroscopy (EDS) elemental mappings were examined using an SEM sample. The SEM image revealed that elemental Hf (white) and O (green) particles were evenly distributed throughout the particles, as shown in Figure 2d. The crystalline structure of the HfO_2_ NPs was examined by using X-ray diffraction (XRD). The XRD pattern obtained was compared to the diffraction pattern of monoclinic HfO_2_ (JCPDS #34-0104, Figure 2e) and found to closely resemble it.

### HfO_2_ NPs as Sonosensitizers

The functionality of the as-synthesized HfO_2_ NPs in the role of sonosensitizers, triggering the production of ROS such as •OH and singlet oxygen (^1^O_2_), was assessed upon exposure to US. In this investigation, terephthalic acid (TA) and methylene blue (MB) were utilized as probes.

TA is a well-known fluorescent probe that reacts with •OH to produce 2-hydroxyterephthalic acid, which emits fluorescence at 420–430 nm.^25^ The results showed that the groups treated with US alone or HfO_2_ NPs (HfO_2_) alone did not yield a detectable fluorescence signal, while the group of the US irradiation of HfO_2_ NPs (HfO_2_+US) resulted in a strong fluorescence signal, indicating that a substantial amount of •OH was generated (Figure 2f). The amount of •OH generated from HfO_2_+US increased with an increasing US irradiation duration.

MB, on the other hand, can be bleached by a wide range of radicals, including ^1^O_2_.^26^ In aqueous solutions, the absorption spectrum of MB typically shows two distinct peaks at around 610 and 670 nm, respectively.^27^ The experimental results showed that the US irradiation of HfO_2_ NPs (HfO_2_+US) caused a significant increase in the degree of MB bleaching compared to the control groups (untreated, US alone, and HfO_2_ alone, Figure 2g). This increase was attributed to the generation of ^1^O_2_ from HfO_2_+US; with an increase in US irradiation time, the amount of ^1^O_2_ generated increased significantly.

The outcomes outlined above strongly indicate the potential of HfO_2_ NPs to serve as inorganic sonosensitizers, a characteristic possibly attributed to their ability to generate high-energy electrons and holes upon activation by US. This inherent capability makes them likely to interact effectively with water and oxygen molecules in their environment, facilitated by the substantial band gap (5.7 eV) of HfO_2_,^28^ subsequently promoting the production of ROS, such as •OH and ^1^O_2_.^29,30^ The resulting ROS can cause oxidative damage to cellular components, leading to cell death.

### Cellular Uptake and Cytotoxicity

SDT, a ROS-based cancer treatment that uses US-triggered sonosensitizers to produce highly toxic ROS, is a promising therapy for killing cancer cells. In order to assess the efficiency of HfO_2_ NPs as sonosensitizers for killing cancer cells, an *in vitro* study using CT26 cells, a colorectal adenocarcinoma cell line, was conducted. The cellular uptake of HfO_2_ NPs and their cytotoxicity toward CT26 cells without US irradiation were first investigated.

**Figure 3:**
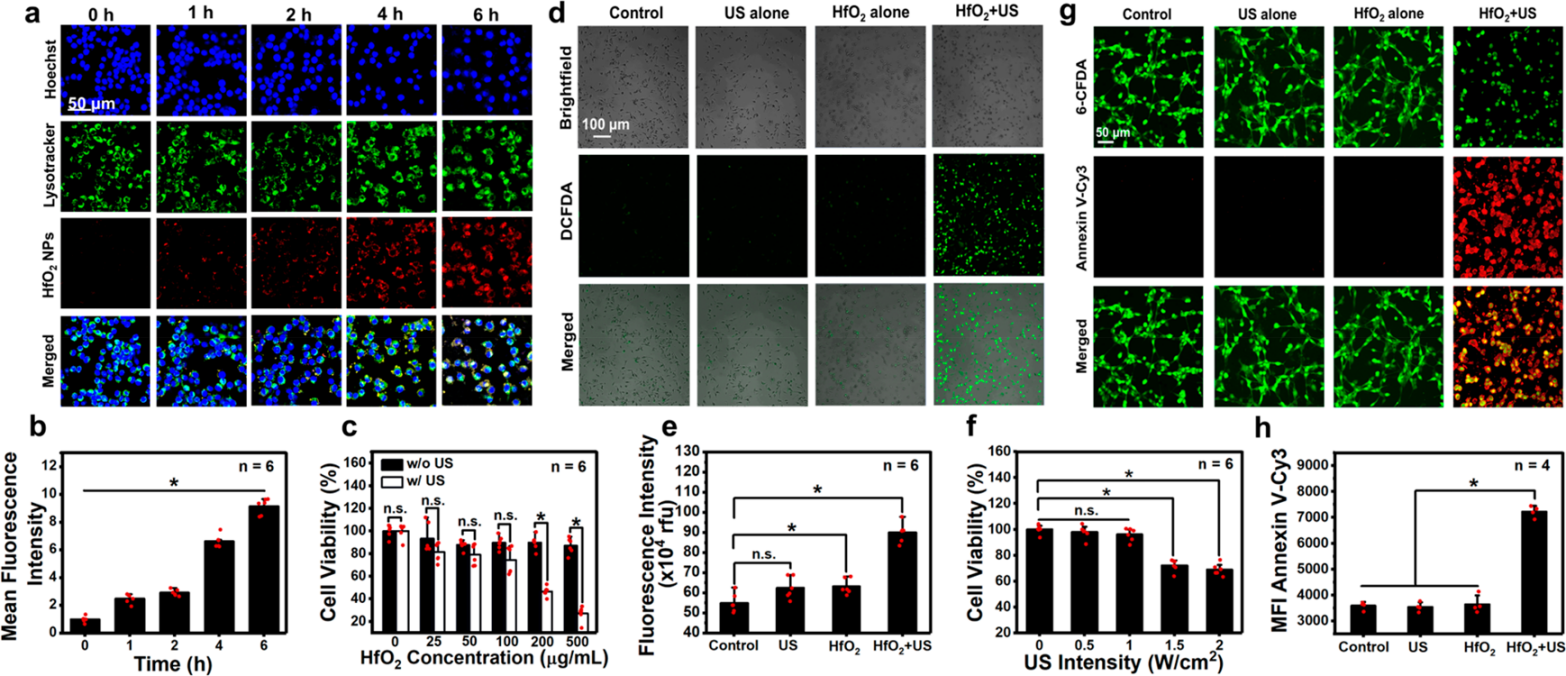
Results of *in vitro* sonodynamic therapy. (a) CLSM images and (b) associated mean fluorescence intensities, showing levels of HfO_2_ NP endocytosis in CT26 cells across different time intervals. (c) Cytotoxicity profiles of HfO_2_ NPs at different concentrations, with (w/) or without (w/o) US irradiation. (d) CLSM images and (e) quantitative assessments of ROS levels in CT26 cells under distinct treatment conditions. (f) Cell viabilities following exposure to US at corresponding power intensities. (g) CLSM images depicting apoptosis of CT26 cells in different treatment groups. (h) Results of flow cytometry analysis showing CT26 cell apoptosis in different treatment groups. Each red dot represents an individual observation. *: statistically significant (*P* < 0.05); n.s.: not statistically significant. US parameters: 1.0 W/cm^2^, 3.0 MHz, 10 min, and 50% duty cycle.

In the cellular uptake study, CT26 cells were incubated with HfO_2_ NPs that had been prelabeled with Alexa Fluor 633 in order to observe their cellular uptake. Confocal laser scanning microscopy (CLSM) images (Figure 3a) revealed that fluorescence was clearly observed within CT26 cells and that the intracellular fluorescence intensity increased over time (Figure 3b), suggesting that HfO_2_ NPs can be effectively taken up by the cells. The cytotoxicity study showed that HfO_2_ NPs had minimal toxicity toward CT26 cells, even at concentrations of up to 500 μg/mL. This was determined by comparing the viability of treated cells with untreated cells and finding that approximately 90% of the cells remained viable (Figure 3c).

To visualize the ROS generated in CT26 cells, 2′,7′-dichlorofluorescin diacetate (DCFDA) was employed, and cell examination was conducted using CLSM. Notably, the fluorescence intensity in CT26 cells incubated with HfO_2_ NPs under US irradiation (HfO_2_+US) far exceeded that of cells treated without US irradiation (HfO_2_ alone) or with US alone, indicating a significant production of ROS *in situ* during HfO_2_+US treatment (Figures 3d and 3e).

In this study, a commonly utilized frequency of 3.0 MHz was employed for US treatment, lasting for 10 min. This frequency falls within the nonthermal range of US frequencies, which is widely applied in the field of SDT.^31^ A low-intensity US of 1.0 W/cm^2^ was chosen to maintain a reasonable cell viability (>90%), as depicted in Figure 3f. While minimal toxicity was observed with HfO_2_ NPs at a concentration of 200 μg/mL without US exposure (HfO_2_ alone), a significant decrease in cell viability (approximately 50%) was observed when the cells were exposed to US (HfO_2_+US, Figure 3c).

The cytotoxicity of the TPU-HfO_2_ composite film was explored by using NIH/3T3 cells from a mouse skin fibroblast cell line; untreated cells were used as a control. Cell viabilities in the test group were comparable to that of the untreated control (*P* > 0.05, Figure S3), suggesting that the film did not demonstrate significant toxicity.

Studies have indicated that SDT can cause apoptotic cell death through the activation of direct sonochemical and subsequent redox reactions.^32^ The results obtained from the analysis of the annexin V-Cy3 (red emission) and 6-CFDA (green emission) double staining^33^ indicated a higher proportion of apoptotic cells, as identified by annexin V-Cy3-positive in CT26 cells upon exposure to HfO_2_+US (Figure 3g). This apoptosis was likely triggered by the intracellularly generated ROS (Figures 3d and 3e), whereas the control groups were mostly viable (annexin V-Cy3-negative, 6-CFDA-positive). Additionally, flow cytometry analysis revealed an increased number of CT26 cells undergoing apoptosis, as evaluated by annexin V-Cy3 labeling, following exposure to HfO_2_+US compared to the control groups (Figure 3h). These findings demonstrate the effective sensitization of cancer cells to US-induced ROS generation and the promotion of cell death by HfO_2_ NPs, highlighting their potential as efficient sonosensitizers for SDT.

### Characteristics of TPU-HfO_2_ Composite Film

TPU, a highly versatile dielectric elastomer material, has the capacity to adapt to the contours of the skin. This distinctive property makes it highly promising for a wide range of on-body applications, particularly in the field of flexible and wearable strain sensors that incorporate electronic components.^34^

In this study, TPU-HfO_2_ composite films were prepared by incorporating HfO_2_ NPs (0, 1, 5, 10, or 15% w/v) into the TPU solution at different concentrations (1, 5, 10, 15, or 20% w/v). To achieve a uniform dispersion, a combination of ultrasonic treatment and continuous stirring was employed to incorporate the HfO_2_ NPs into the TPU solution. The solution casting method was then used to fabricate composite films with a target thickness of approximately 150 μm. For strain sensors to conform well to the skin, film thicknesses ranging from hundreds to thousands of micrometers are typically required.^35^ It is worth noting that the TPU solution with a concentration of 1% (w/v) did not form a film due to its low viscosity. On the other hand, the TPU solution with a concentration of 20% w/v exhibited excessive viscosity, which hindered the effective dispersion of the HfO_2_ NPs.

**Figure 4:**
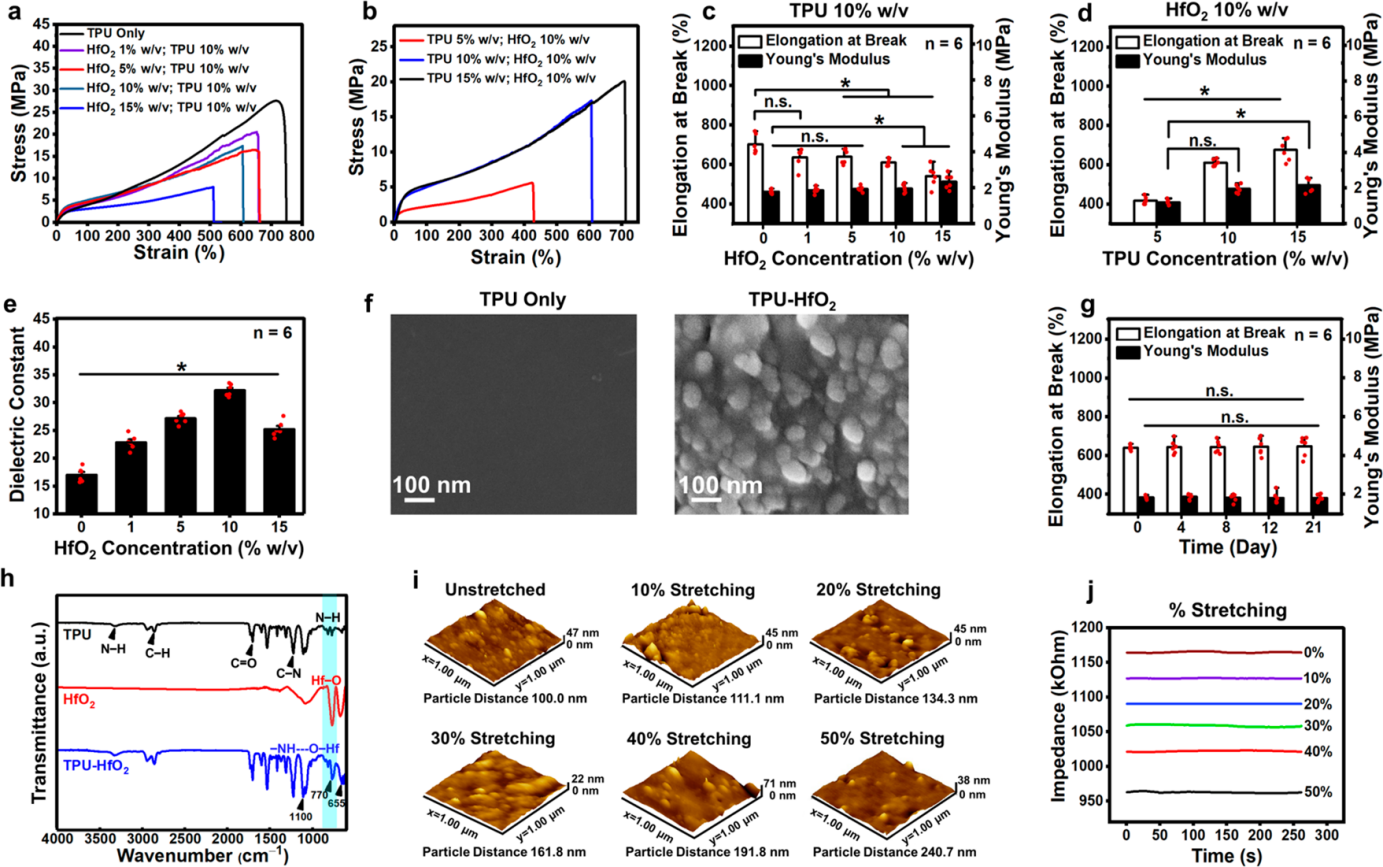
Characteristics of TPU-HfO_2_ composite films. (a) Stress–strain curves of TPU film and TPU-HfO_2_ composite films, fabricated using TPU at a concentration of 10% w/v and varying concentrations of HfO_2_ NPs. (b) Stress–strain curves of TPU-HfO_2_ composite films prepared with HfO_2_ NPs at a concentration of 10% w/v and different concentrations of TPU. (c) Values of elongation at break and Young’s modulus of TPU film and TPU-HfO_2_ composite films prepared with TPU (10% w/v) and different concentrations of HfO_2_ NPs. (d) Values of elongation at break and Young’s modulus of TPU-HfO_2_ composite films fabricated using HfO_2_ NPs (10% w/v) with different concentrations of TPU. (e) Dielectric constants of TPU film and TPU-HfO_2_ composite films across different HfO_2_ concentrations, conducted at 100 kHz frequency. (f) SEM micrographs displaying cross sections of TPU film and TPU-HfO_2_ composite film. (g) Values of elongation at break and Young’s modulus of an optimized TPU-HfO_2_ composite film subjected to consistent strain over durations of 0, 4, 8, 12, and 21 days. (h) FTIR spectra of TPU film, HfO_2_ NPs, TPU-HfO_2_ composite film. (i) Representative AFM images depicting distributions of HfO_2_ NPs and their average distances in unstretched and stretched TPU-HfO_2_ composite films. (j) Electrical impedances of TPU-HfO_2_ composite films in unstretched and stretched conditions, recorded at an output voltage of 100 mV and a frequency of 20 kHz. Each red dot represents an individual observation. *: statistically significant (*P* < 0.05); n.s.: not statistically significant.

To gain a comprehensive understanding of the material properties, the stress–strain behavior of both the pure TPU film and its TPU-HfO_2_ composites were examined. Figures 4a and 4b illustrate the stress–strain curves for these materials, while Figures 4c and 4d provide a direct comparison of their Young’s modulus and elongation at break. The pure TPU film, with a TPU concentration of 10% w/v, displayed a Young’s modulus of 1.8 MPa and an elongation at break of approximately 700% strain. It is important to note that the Young’s modulus of human skin typically falls within the range of 0.1 kPa–2.0 MPa, while exhibiting a stretchability exceeding 70%.^36^

The incorporation of HfO_2_ NPs into the TPU film resulted in a relative increase in Young’s modulus and a decrease in elongation at break. Additionally, increasing the TPU concentration led to increases in both Young’s modulus and elongation at break. Notably, the Young’s moduli of the TPU-HfO_2_ composite films, with varying concentrations of HfO_2_ NPs and TPU, did not significantly differ from that of human skin. Furthermore, all of these composite films exhibited elongation at breaks exceeding 400%, a value substantially surpassing that of human skin. This evident contrast emphasizes their notable potential for designing stretchable strain sensors specifically suited for on-body applications.

TPU is well-known for its high dielectric constant. In this study, the dielectric constant of the TPU film was found to be further enhanced upon incorporation of HfO_2_ NPs (Figure 4e). As the concentration of HfO_2_ NPs increased in the TPU film, the dielectric constant of the resulting TPU-HfO_2_ composite film exhibited a rising trend, reaching its maximum value of 32.2 at 100 kHz with a concentration of 10% w/v. This optimized formulation, consisting of both HfO_2_ NPs and TPU at a concentration of 10% w/v, was selected for fabricating the TPU-HfO_2_ composite film intended for use as the proposed DE strain sensor in subsequent applications. SEM was used to analyze the distribution of HfO_2_ NPs within the composite film. The resulting SEM micrograph provided a clear visualization of the uniform dispersion of HfO_2_ NPs throughout the cross-sectional area of the TPU film (Figure 4f).

The optimized TPU-HfO_2_ composite film is designed to be placed on top of a tumor, serving as a DE strain sensor for monitoring its growth and regression over an extended period. It is crucial that the film maintains stable mechanical properties without significant deterioration during the investigation. The mechanical stability of the TPU-HfO_2_ composite film was evaluated by subjecting it to a 35% strain, simulating conditions similar to a tumor with a size of 1200 mm^3^ (see the *In Vitro* Tumor Volume Measurements section for reference). This dimension corresponds to the maximum allowable tumor size in mice based on ethical considerations.^37^

Over a period of 21 days, the film exhibited minimal changes in both Young’s modulus and elongation at break (*P* > 0.05, Figure 4g). TPU films are widely recognized for their exceptional durability and ability to endure deformation and stress with minimal degradation.^38^ These results indicate excellent mechanical stability, ensuring the long-term performance and reliability of the TPU-HfO_2_ composite film as a DE strain sensor for the continuous monitoring of the progression of tumors.

To explore potential specific interactions between TPU and the HfO_2_ NPs within the composite film, Fourier transform infrared spectroscopy (FTIR) was employed. Figure 4h illustrates the FTIR spectra of the TPU film, HfO_2_ NPs, and TPU-HfO_2_ composite. TPU displayed prominent peaks at 3330 cm^–1^, denoting N–H stretching, 1700 cm^–1^, representing C=O stretching, and 1220 cm^–1^, indicating C–N stretching vibrations attributed to the urethane functional groups (−NH–(C=O)–O−). Of particular interest is the FTIR spectrum of the TPU-HfO_2_ composite, which revealed distinct peaks at 1100, 770, and 655 cm^–1^. These peaks indicate the presence of Hf–O bonds (HfO_2_ NPs) within the TPU composite material. Significantly notable is the range of heightened peak intensity spanning from 880 to 726 cm^–1^, strongly suggesting a robust intermolecular hydrogen bonding interaction facilitated between the amide (−NH) group of TPU and the oxygen (−O) atoms of the HfO_2_ NPs.

An additional experiment was carried out using FTIR to examine potential chemical changes in the TPU-HfO_2_ composite film when subjected to stretching conditions. FTIR is a useful tool for detecting chemical alterations that might indicate degradation due to exposure to environmental factors.^39^ Comparing the FTIR spectrum of the composite film before and after subjecting it to a 35% strain for 21 days, there were no observable shifts or the emergence of new absorption peaks (Figure S4). This result indicates the film’s chemical durability during the entire stretching period. Furthermore, the intermolecular hydrogen bonding interactions between the amide (NH) group of TPU and the oxygen (−O) atoms of the HfO_2_ NPs remained undisturbed. This supports the film’s structural integrity and its ability to prevent particle release throughout the entire duration of the experimental study.

When there are changes in tumor volume, the DE strain sensor experiences mechanical deformation or strain, which leads to alterations in the distances among the embedded HfO_2_ NPs within the TPU film. To investigate these changes, atomic force microscopy (AFM) was utilized to examine the average distance between the particles under different mechanical strains. Figure 4i demonstrates that before stretching the initial distribution of HfO_2_ NPs in the TPU film was reasonably even. However, upon stretching the film, a noticeable increase in the average distance among the particles was observed, which directly corresponds to the applied mechanical strain.

In response to changes in the distances among the HfO_2_ NPs, the capacitance of the TPU-HfO_2_ composite film experienced adjustments. These alterations in capacitance led to fluctuations in the electrical impedance of the film (Figure 4j). This phenomenon could potentially provide significant insights into the degree of deformation or strain induced by factors such as variations in tumor volume.

### *In Vitro* Tumor Volume Measurement

The capability of the TPU-HfO_2_ composite film to measure tumor volume and track its temporal growth and regression was evaluated *in vitro* by using simulated tumors of varying sizes. The assessment of tumor volume changes is a common practice in determining treatment initiation, dosing optimization, and evaluating treatment effectiveness. However, the specific size at which a tumor is deemed suitable for treatment can differ, influenced by factors such as cancer type, location, and established medical protocols.^40,41^ In this study, a tumor volume threshold of 100 mm^3^ was selected as a criterion to initiate treatment. This choice was made based on the understanding that tumors reaching this size might indicate a critical point, signaling a potential shift toward increased aggressiveness and resistance to the body’s immune responses.^42^ Therefore, a tumor volume of 100 mm^3^ serves as an indication for intervention as it implies a transition to a stage where therapeutic actions become imperative for effective management.

**Figure 5:**
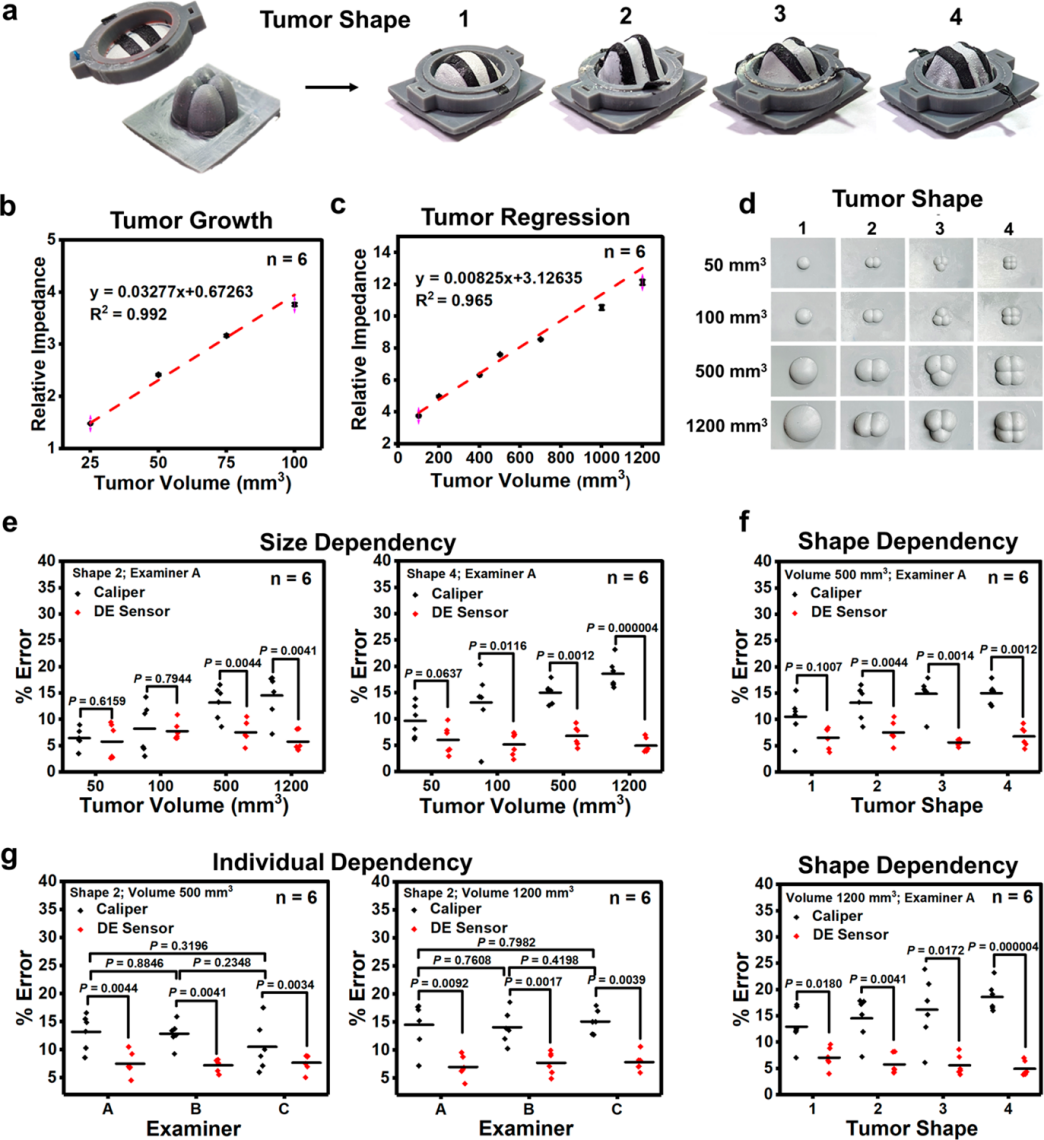
Results of *in vitro* tumor volume measurements. (a) Photographs of 3D-printed tumor models and rings. The action of pressing downward on the plastic ring causes the TPU-HfO_2_ film within it to deform, conforming to the distinct lobes of the simulated tumor model. (b) Calibration curve for tumor growth. (c) Calibration curve for tumor regression. (d) Photographs of 3D-printed simulated tumor models exhibiting a variety of shapes and sizes. (e) Error percentages in tumor volume measurement for varying sizes of shape 2 and shape 4 simulated tumor models, as measured by both a caliper and the DE strain sensor. (f) Error percentages in tumor volume measurement for diverse tumor shapes, with volumes of 500 and 1200 mm^3^, utilizing both a caliper and the DE strain sensor. (g) Error percentages in tumor volume measurement for shape 2 simulated tumor models with volumes of 500 and 1200 mm^3^, as examined by three different individuals. Each red dot represents an individual observation.

To quantify the volume, the simulated tumor was positioned beneath a TPU-HfO_2_ composite film featuring two carbon-tape electrodes serving as a DE sensor. The DE sensor was secured using a plastic ring matching the diameter of the simulated tumor. Upon firmly pressing the ring downward, the composite film was stretched (Figure 5a). The electrical impedances prior to stretching (*Z*_0_) and after stretching (*Z*) were measured using an ELITE EIS data recorder at a frequency of 20 kHz and a voltage of 100 mV. Frequencies of around 20 kHz in the applied electrical field are commonly utilized for on-body sensing applications, facilitating the detection of electrical impedances. The use of low voltage reduces power consumption and ensures safe operation without inducing significant tissue heating or other adverse effects.^43^

To replicate tumor growth scenarios, simulated tumors ranging in sizes of 25, 50, 75, and 100 mm^3^ were generated. For the simulation of tumor regression, a spectrum of simulated tumor sizes spanning from 100 to 1200 mm^3^ was employed. These artificial tumors were meticulously fabricated by using advanced 3D printing technology. To establish a reliable means of quantifying both tumor growth and regression dynamics, calibration curves were meticulously devised. These curves were constructed by plotting the relative impedance [(*Z*_0_ – *Z*)/*Z*_0_] values against the corresponding simulated tumor volumes. Figures 5b and 5c provide visual representations showcasing the alignment of regression lines from these calibration curves with actual data points. This alignment distinctly underscores the robust relationship between the relative impedance and tumor volume. The correlation coefficients (*R*^2^) further affirm this correlation, measuring at 0.992 for tumor growth in Figure 5b, and 0.965 for tumor regression in Figure 5c.

Notably, the developed TPU-HfO_2_ DE sensor demonstrates exceptional precision in measurements, reaching its accuracy even when dealing with minute tumor volumes such as 25 mm^3^. This achievement stands in contrast to the clinical realm of CT scanning, where the smallest detectable tumor typically hovers around 200 mm^3^.^44^

The conventional technique for measuring subcutaneous tumor volumes involves using an external caliper. Nevertheless, this method is subjective and prone to errors influenced by tumor size, shape, and the individual conducting the measurement.^45,46^ To comprehensively assess and facilitate a meaningful comparison between the measurement accuracy of the TPU-HfO_2_ composite film (DE sensor) and the caliper, a range of simulated tumors were employed. The simulated tumors exhibited variations in size (50, 100, 500, and 1200 mm^3^) and featured distinct hemispheric shapes: one lobe (shape 1), two lobes (shape 2), three lobes (shape 3), or four lobes (shape 4) (Figure 5d). To ensure a comprehensive assessment, measurements were conducted by three different individuals.

When the accuracy of the caliper measurements for tumor volume was evaluated, a noticeable trend emerged: the error percentage in measurements tended to increase as the tumor size increased. This trend was consistently observed, ranging from 7% (shape 2) to 10% (shape 4) for a 50 mm^3^ tumor, and from 15% (shape 2) to 18% (shape 4) for a 1200 mm^3^ tumor (Figure 5e). As the tumor size enlarges, accurately gauging its dimensions—length, width, and height—using a caliper becomes progressively challenging due to irregular shapes.^47,48^ Such inaccuracies in measurements can lead to inflated error percentages when computing tumor volume. In contrast, the precision of the DE sensor remained relatively steady, with errors consistently falling within the range of 5% to 8% across the entire range of investigated tumor sizes.

Tumors often exhibit heterogeneous growth patterns, frequently deviating from the ideal spherical shape.^49^ As the tumor volume increases, these heterogeneous characteristics become more pronounced, posing challenges for accurately capturing boundaries and dimensions using caliper measurements. This trend is illustrated in Figure 5f, where the caliper method for volume determination exhibited escalating inaccuracies as irregular tumor shapes were encountered. In contrast, the DE sensor consistently maintained a stable error percentage. For example, with a simulated tumor featuring one (four) hemispheric lobe(s) and a volume of 1200 mm^3^, error percentages stood at 13% (18%) for the caliper method and 7% (6%) for the DE sensor.

The accuracy of the caliper method is reliant on the technique utilized by each individual performing the measurements. Variances in measurement techniques introduced by different individuals can result in varying errors in the assessment of the tumor volume. The observations, depicted in Figure 5g, highlight that the interindividual variation in error percentages attributed to the DE method (around 7%) is notably lower compared to that arising from the caliper method (ranging from 10% to 15%).

These results highlight the significant advantage of the DE sensor over traditional caliper methods when it comes to delivering precise volumetric measurements. This advantage becomes particularly evident as tumor size and shape irregularity increase. This enhanced performance can be attributed to the flexibility and stretchability of the developed TPU-HfO_2_ composite film, allowing it to snugly conform to the surface of the simulated tumor, regardless of its dimensions and shape (Figure 5a). This characteristic equips the sensor with high sensitivity and accuracy in its role as a DE strain sensor.

### Dose-Dependent Treatment Effectiveness of HfO_2_+US

The subsequent phase of the study involved assessing the *in vivo* efficacy of HfO_2_ NPs as sonosensitizers for SDT. Prior to validating the antitumor potential of HfO_2_+US, the investigation examined the impact of various doses of HfO_2_ NPs on mice with subcutaneous CT26 tumors. The treatment schedule, outlined in Figure S5a of the Supporting Information, followed a sequence that began with tumor sizes of around 100 mm^3^, as assessed using a caliper, on days 0, 1, and/or 2 (single dose, two doses, or three doses).

Each intratumoral dose consisted of 200 μg/mL of HfO_2_ NPs (0.1 mL), suspended in saline, and was coupled with US irradiation (1.0 W/cm^2^) for a duration of 10 min. As a comparison, mice that solely received saline served as the untreated control group. On the day when the tumor volume of the untreated group reached 1200 mm^3^, the treated tumors were harvested for further analysis. Subsequently, these tumors were subjected to photography, and their volumes and weights were measured to facilitate a comprehensive analysis.

As depicted in Figures S5b–S5d, noticeable trends emerged from the results. Mice subjected to a three-dose regimen of HfO_2_+US demonstrated a substantially more pronounced reduction in both tumor volumes and weights compared to counterparts that received either single or two doses of HfO_2_+US (*P* < 0.05). Furthermore, their body weights exhibited consistent levels across the study duration (*P* > 0.05, Figure S5e). Given these results, the subsequent HfO_2_+US treatment regimen followed a three-dose protocol.

### *In Vivo* Tumor Volume Monitoring

**Figure 6:**
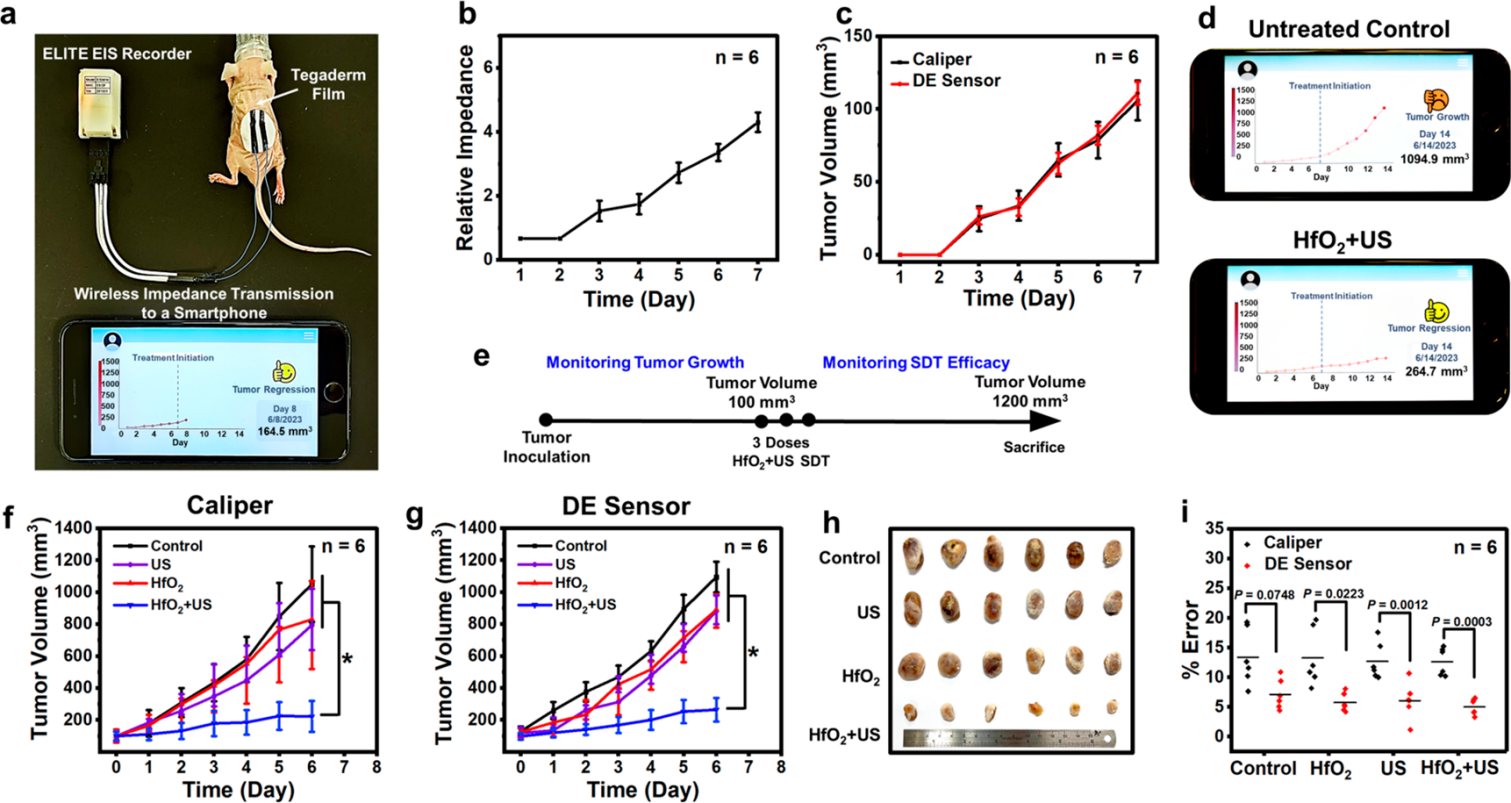
Results of *in vivo* tumor volume monitoring before and after HfO_2_+US-based SDT. (a) Photograph depicting the experimental setup for monitoring tumor volume changes in a test mouse. The DE strain sensor is securely attached to a subcutaneous tumor using a Tegaderm film and connected to an ELITE EIS recorder. Results of the electrical impedance measurement are transmitted wirelessly to a smartphone. (b) Relative impedances recorded for 7 days prior to the SDT. (c) Comparison of tumor volumes measured by a caliper and the DE strain sensor, with relative impedance values converted using an established calibration curve for temporal tumor growth. (d) Smartphone displays of tumor progressions for untreated mice and mice treated with HfO_2_+US. (e) Schematic time course of establishment of tumor model and subsequent SDT treatment and monitoring tumor dynamics. (f) Tumor volumes measured by a caliper and (g) the DE strain sensor for test mice subjected to various treatments. (h) Photograph of excised tumors of mice in different treatment groups. (i) Error percentages in tumor volume measurements using a caliper or the DE strain sensor in comparison to the tumor volume derived from the water displacement method across all treatment groups. Each red dot represents an individual observation. *: statistically significant (*P* < 0.05). US parameters: 1.0 W/cm^2^, 3.0 MHz, 10 min, and 50% duty cycle.

To track alterations in tumor volume post-treatment, the TPU-HfO_2_ composite film, fitted with two carbon-tape electrodes, was strategically placed onto the inoculated tumor site. To ensure a secure attachment, a transparent Tegaderm adhesive film was employed, meticulously securing the composite film’s perimeter and guaranteeing its smooth adherence to the tumor’s surface. The measurement of relative impedance in relation to tumor volume was carried out using conventional alligator-style lead clips, which connected the ELITE EIS recorder to the two carbon-tape electrodes. The impedance measurements obtained were then wirelessly transmitted to a personal smartphone that was equipped with a data processing application. This setup allowed for the real-time evaluation of the status of tumor progression in the mouse model with a subcutaneous tumor (Figures 1 and 6a; Video S1).

It is evident that prior to the commencement of treatment, there was a consistent and notable increase in the relative impedance [(*Z*_0_ – *Z*)/*Z*_0_] over time (Figure 6b). The tumor volumes, derived from the relative impedance values using the well-established calibration curve for temporal tumor growth (Figure 5b), aligned closely with the volumes obtained through caliper measurements (*P* > 0.05, Figure 6c). This alignment underscores the effectiveness of the developed TPU-HfO_2_ composite film as a DE strain sensor, showcasing its capability to monitor alterations in the tumor volume.

Importantly, as the tumor volume approached the critical threshold of 100 mm^3^, a distinctive alert signal was activated on the patient’s smartphone (Figure 6d; Videos S2 and S3). This pivotal feature served as a notification mechanism, simultaneously alerting both the patient and the remote medical staff. The signal marked the beginning of the three-dose treatment regimen involving HfO_2_+US (Figure 6e). To provide a basis for comparison, the control groups consisted of untreated mice and mice that underwent treatment protocols involving either HfO_2_ alone or US alone.

Figures 6f and 6g illustrate a consistent trend in the observed changes in tumor volumes, determined either by the caliper method or derived from DE sensor-detected impedances using the established calibration curve for temporal tumor regression (Figure 5c). Despite the differences in the measurement techniques, the overall profile of tumor volume progression over time remained comparable. This result underlines the reliability and accuracy of the DE sensor measurements in effectively capturing the tumor’s growth dynamics. In the case of individual treatments involving HfO_2_ alone or US alone, there was no significant inhibition in tumor growth observed (Figures 6f–6h, *P* > 0.05), compared with the untreated control group. In contrast, mice treated with HfO_2_+US exhibited a substantial impediment in tumor development (*P* < 0.05). This finding underscores the efficacy of the ROS generated *in situ* by HfO_2_ NPs at the site of US irradiation (as demonstrated *in vitro* in Figures 3d and 3e) in achieving effective tumor therapy.

The use of the developed DE sensor allows for real-time visualization of treatment effectiveness through the direct monitoring of tumor volume changes, conveniently accessible on the patient’s smartphone (Figure 6d). Significantly, even when the tumor reached its maximum observed volume of 1200 mm^3^, the DE strain sensor stretched by approximately 35% without applying an excessive force that might disrupt or potentially rupture the tumor during the entire study. This can be attributed to the pliability, flexibility, and lightweight nature of the developed composite film, which collectively function to mitigate potential negative impacts from shear forces. Featuring a user-friendly interface and the ability for continuous and accurate monitoring, this intelligently designed system has the potential to noninvasively illustrate the real-time progression of tumor volume.

At the point where the tumor volume in the untreated control group reached around 1200 mm, signifying completion of the treatment regimen, the experimental mice were euthanized. Subsequently, the treated tumors were carefully excised, and reference photographs were captured for documentation purposes (Figure 6h). Before the tumors were extracted from each experimental group, detailed volumetric measurements were conducted using both the caliper and the DE sensor. These measurements were then methodically compared to the harvested tumor volumes determined through a water displacement method, acknowledged for its accuracy and thorough volumetric assessment.^4^

As depicted in Figure 6i, in comparison to the tumor volume derived from the water displacement method, the error percentages recorded by the DE sensor (averaging around 5 to 10%) consistently exhibited a significant improvement over those obtained by the caliper (ranging from approximately 10 to 20%). The DE strain sensor effectively utilizes its inherent flexibility and stretchability, along with its aligned Young’s modulus to that of the skin tissue, allowing it to promptly react to mechanical deformations caused by changes in tumor volume. This dynamic characteristic equips the DE sensor with the ability to skillfully capture and precisely quantify intricate deformations and irregularities within complex tumor geometries. As a result, the DE sensor method emerges as a more advanced and effective approach, outperforming the capabilities of the caliper technique.

### Treatment Efficacy

**Figure 7:**
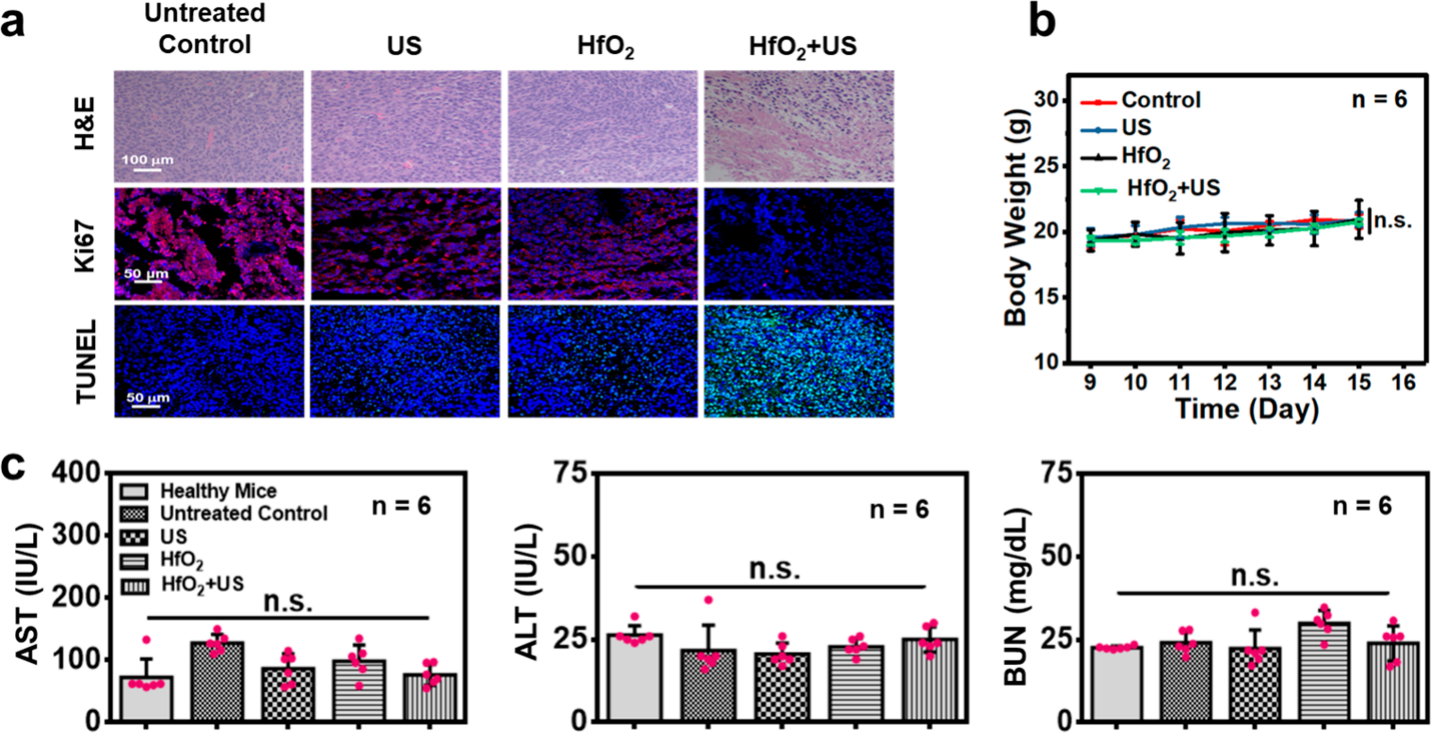
Results of treatment efficacy and biosafety. (a) H&E staining, Ki67 staining, and TUNEL assay of tumor tissues that were retrieved from different treatment groups. (b) Changes in body weights of test mice observed in different treatment groups. (c) AST, ALT, and BUN levels in sera in healthy and treated tumor-bearing mice. Each red dot represents one observation. n.s.: not statistically significant. US parameters: 1.0 W/cm^2^, 3 MHz, 10 min, and 50% duty cycle.

To gain deeper insights into the antitumor efficacy of each test group, the harvested tumor tissues underwent comprehensive histological analyses, encompassing hematoxylin and eosin (H&E) staining, Ki67 staining, and terminal deoxynucleotidyl transferase dUTP nick end labeling (TUNEL) assay. Figure 7a illustrates the outcomes, showcasing how treatment with HfO_2_+US yielded a significant reduction in the number of tumor cells (H&E stain) compared to the control groups. Moreover, this treatment resulted in a reduced proportion of proliferating cells (Ki67 stain) and an elevated presence of apoptotic cells (TUNEL assay). TUNEL assay is renowned for its capacity to detect DNA fragmentation, a characteristic feature of apoptotic cells that can be induced by SDT using HfO_2_+US.^50^ This marker is a valuable tool for assessing the impact of SDT on tumor cells, which is also supported by the *in vitro* apoptosis assay (Figures 3g and 3h).

To comprehensively assess the biosafety of each test formulation, additional experiments were conducted, involving the examination of alterations in the body weights and blood chemistry of the test mice as well as the analysis of histological sections from their primary organs. In this regard, no discernible decline in body weight was observed within any of the investigated groups (*P* > 0.05, Figure 7b). The levels of serum aspartate aminotransferase (AST) and alanine aminotransferase (ALT), serving as indicators of hepatic function, along with blood urea nitrogen (BUN), a marker of renal function, were all found to be well within the normal range (*P* > 0.05, Figure 7c). This cumulative data strongly suggests the lack of observable hepatic or renal dysfunction. Furthermore, upon the evaluation of the histological sections from the main organs stained with H&E, no apparent inflammatory infiltrates or histological abnormalities were observed (Figure S6). These combined observations provide compelling evidence of the effective and safe role played by intratumorally administered HfO_2_ NPs. This confirmation reinforces their status as potent and safe sonosensitizers.

Developing a smart sensor capable of noninvasive, precise, and continuous real-time monitoring of tumor growth and regression has presented a significant challenge. Strain sensors are instrumental devices employed to quantify the deformation or strain experienced by objects upon being subjected to external forces. They find versatile applications across biomedical domains such as biomechanics, robotics, and on-body healthcare monitoring.^51,52^ While common strain sensors, including piezoelectric, capacitive, resonant, and thin-film variants made from metals or semiconductor materials, are recognized for their heightened sensitivity, they do come with inherent limitations.^53^ These sensors are primarily fixed-directional and capable of measuring strain exclusively along specific axes. Moreover, their flexibility and stretchability are frequently confined, suggesting opportunities for further advancement to overcome these constraints.

DE strain sensors have garnered significant interest in recent times, owing to their simple operation and diverse applicability. Traditional DE strain sensors typically consist of a dielectric elastomer film layer, such as silicone rubber, positioned between two layers of electrodes made from either rigid metals or compliant conductive polymers.^54,55^ Nonetheless, the application of silicone rubber as the dielectric elastomer film layer is constrained by its limited strain sensing performance, mainly attributed to its low dielectric constant (2.7).^53^ Furthermore, although these electrodes may exhibit commendable electrical properties, an inherent challenge arises from the dissimilar levels of flexibility and stretchability exhibited by the central dielectric elastomer film in contrast to the top and bottom electrode layers.^56^ This issue can lead to complications in specific applications, particularly those requiring significant deformations or the adaptation to the contours of soft, curvilinear tissues, thus influencing the precision and reliability of these strain sensors.

In a recent study, an innovative strain sensor was introduced, consisting of a flexible polymeric film coated with gold, designed for tracking tumor progression by monitoring changes in its electrical resistance.^1^ It was found that as tumor progression occurred, the resistance values in the sensor increased, aligning with the data obtained from caliper and bioluminescent imaging. However, it is essential to acknowledge that the sensor provided resistance values rather than direct measurements of the tumor volume. This absence of direct tumor volume measurements constrained our ability to conduct comprehensive and precise volume comparisons.

Conversely, the DE strain sensor developed in this study is centered around a single-layered TPU-HfO_2_ composite film. With its carefully tailored Young’s modulus (Figures 4c and 4d), it seamlessly matches the mechanical properties of the skin. Its inherent flexibility enables it to easily adapt to irregularly shaped tumor surfaces (Figure 5a). Functioning akin to a deformable capacitor, this DE strain sensor displays shifts in electrical impedance in response to deformation in all dimensions (Figures 5b and 5c), providing a direct reflection of changes in the tumor volume underneath (Figures 6d and 6g). Furthermore, the strain sensing capabilities of the developed TPU-HfO_2_ composite film are enhanced by its high dielectric constant (32.2, Figure 4e), establishing it as an advanced variant of the DE strain sensor.

Through the seamless integration of this strain sensing platform with the ELITE EIS data recorder (Figure 6a), the strength of this monitoring system lies in its capacity to effortlessly transmit real-time data on tumor volume progression to both the patient’s personal smartphone (Figure 6d) and the remote medical staff, facilitated by a WI-FI system. This dynamic exchange of data empowers timely interventions guided by expert assessments.

## Conclusions

Collectively, the aforementioned findings provide compelling evidence that the synthesized HfO_2_ NPs can effectively and safely serve as sonosensitizers capable of eradicating cancer cells under US irradiation. The wearable TPU-HfO_2_ DE strain sensor developed demonstrates exceptional precision in measurements, maintaining accuracy even when dealing with minute tumor volumes, as evidenced by a significantly lower error percentage (averaging around 5 to 10%) compared to those obtained by the caliper (ranging from approximately 10 to 20%) in *in vivo* experiments. Furthermore, its proficiency in monitoring tumor progression aligns well with the demands of personalized medicine. Consequently, they emerge as a highly promising candidate for the next generation of device-based cancer care in the field of telemedicine.

## Materials and Methods

### Materials

Hafnium(IV) chloride (HfCl_4_), sodium hydroxide (NaOH), terephthalic acid (TA), and methylene blue (MB) were procured from Sigma-Aldrich (St. Louis, MO, USA). TPU (BASF Elastollan 1185A) was acquired from BASF Co., Ltd. (Ludwigshafen, Germany). Phrozen Aqua 4K 3D Printing Resin (Young’s modulus = 1037 MPa) utilized in the 3D-printed tumor model was obtained from Phrozen Tech Co., Ltd. (Hsinchu, Taiwan). The Alexa Fluor 633 NHS ester was obtained from Thermo Fisher Scientific (Waltham, MA, USA). DAPI was obtained from Invitrogen (Carlsbad, CA, USA). The CT26 (ATCC CRL-2638) cells were acquired from the American Type Culture Collection (Manassas, VA, USA). The mouse skin fibroblast cell line (NIH/3T3) was procured from the Bioresource Collection and Research Center, Food Industry Research and Development Institute (Hsinchu, Taiwan). Cell culture reagents were obtained from Gibco (Grand Island, NY, USA). All other chemicals and reagents used were of analytical grade.

### Synthesis and Characterization of HfO_2_ NPs

To synthesize HfO_2_ NPs, a solution containing a mixture of 50 mM HfCl_4_ and 3 M NaOH was prepared by using 88 mL of deionized (DI) water. After stirring, the solution was introduced into a sealed 100 mL Teflon-lined stainless-steel container. This container was then heated to 120 °C for 20 h. The resulting HfO_2_ NPs were obtained through centrifugation, followed by three rounds of cleaning with DI water. Afterward, they were dried at 60 °C overnight and calcined at 700 °C for 1 h.

The chemical composition and valence state analyses of the as-synthesized HfO_2_ NPs were conducted using XPS (ULVAC-PHI HRXPS, Ulvac-PHI Inc., Chigasaki, Japan). The morphological structure was examined using SEM (JSM-5600, JEOL Technics, Tokyo, Japan), and elemental composition was mapped using EDS. The dimensions of the HfO_2_ NPs were determined through SEM and analyzed by using ImageJ software. The zeta potential in DI water was determined through DLS (Zetasizer, 3000 HS, Malvern Instruments, Worcestershire, UK). The dielectric properties were assessed with an Impedance Analyzer (Agilent 4294A, Agilent Technologies, Santa Clara, CA, USA) at room temperature, operating at 20–100 kHz frequency. The dielectric constant (κ) was derived using the parallel plate capacitor formula, κ = *Ct*/ε_0_*A*, where *C* represents capacitance, *t* represents capacitor thickness, ε_0_ signifies the vacuum dielectric constant, and *A* stands for electrode area.^57^ Crystalline structure analysis was performed by using an X-ray diffractometer (Cu Kα radiation, XRD-6000, Shimadzu, Tokyo, Japan).

### Evaluation of HfO_2_ NPs as Sonosensitizers

The potential of HfO_2_ NPs to function as sonosensitizers under US irradiation, generating •OH or ^1^O_2_ was evaluated. To elaborate, 4.75 mM HfO_2_ NPs was uniformly dispersed in a 4 mL solution containing TA (5 mM) or MB (0.03 mM). This mixture was stirred for 2 h. Subsequently, the combined solution underwent US irradiation (3 MHz, 1 W/cm^2^, Sonopuls 190, Rotterdam, Netherlands) for 10 min. To safeguard against any degradation of TA and MB due to photocatalysis, the entire reaction took place within a dark chamber.^58^ The fluorescence (TA) and absorbance (MB) spectra of the solution were measured at specified intervals using a SpectraMax M5Microplate Reader (Molecular Devices, San Jose, CA, USA).

### Cellular Uptake of HfO_2_ NPs

To visualize the cellular uptake of HfO_2_ NPs, CT26 cells were incubated with Alexa Fluor 633-conjugated HfO_2_ NPs (f-HfO_2_ NPs) at a concentration of 200 μg/mL. The preparation of f-HfO_2_ NPs followed a previously reported procedure.^59^ Briefly, a solution of (3-aminopropyl)triethoxysilane (APTES)-Alexa Fluor 633 was prepared by mixing 3 mL of cyclohexane, 100 μL of APTES, and 0.1 mg of Alexa Fluor 633, followed by stirring for 24 h. The HfO_2_ NPs (8 mM in 90% ethanol) were mixed with the prepared APTES-Alexa Fluor 633 under rapid stirring for 6 h at room temperature. Subsequently, f-HfO_2_ NPs were collected and separated from unreacted APTES-Alexa Fluor 633. The cells were incubated with f-HfO_2_ for predetermined time intervals (0, 1, 2, 4, or 6 h). Subsequently, the cells were collected, washed with phosphate-buffered saline (PBS), stained with DAPI, and examined using CLSM (Zeiss LSM780, Carl Zeiss, Jena, Germany).

### Cytotoxicity and Intracellular ROS Generation

In this study, CT26 and NIH/3T3 cells were separately cultured in 96-well plates at a density of 1 × 10^4^ cells per well. CT26 cells were exposed to various concentrations of HfO_2_ NPs and then subjected to US irradiation (3 MHz, 1 W/cm^2^, 10 min). Following 24 h of incubation, cell viability was evaluated using a CellTiter-Glo Luminescent Cell Viability Assay Kit (Promega, Madison, WI, USA). For NIH/3T3 cells, the cytotoxicity of the TPU-HfO_2_ film was evaluated through an elution test.^60^ The test composite film was incubated in a medium supplemented with 10% bovine calf serum at 37 °C for 24 h. Subsequently, the cells were treated with the extract from the test film for an additional day. The cell viability was determined using the CellTiter-Glo Luminescent Cell Viability Assay Kit.

For assessing intracellular ROS generation, CT26 cells underwent the same treatment protocol. Post-treatment, the production of ROS within the cells was quantified using DCFDA (25 μM, Abcam, Cambridge, UK). Both qualitative observations and quantitative measurements of ROS were performed using CLSM and a SpectraMax M5 spectrophotometer, respectively.

### Cellular Apoptosis

CT26 cells were cultured overnight in 12-well plates (1 × 10^5^ cells/mL) and subjected to one of the following test formulations; untreated control, US alone, HfO_2_ alone, and HfO_2_+US. Thereafter, the apoptosis of the treated cells was assessed using an APOAC Annexin V-Cy3 Apoptosis detection kit (Sigma-Aldrich, St. Louis, MO, USA). The analysis was performed through CLSM and a flow cytometer (BD Accuri C6, BD Biosciences, San Jose, CA, USA); the data thus obtained were further processed by using FlowJo software (Treestar, Ashland, OR, USA).

### Preparation and Characterization of TPU-HfO_2_ Composite Film

TPU-HfO_2_ composite films were created by integrating HfO_2_ NPs (0, 1, 5, 10, or 15% w/v) into the TPU solution at varying concentrations (1, 5, 10, 15, or 20% w/v). The process began with dissolving TPU pellets in a dimethylformamide (DMF) solvent. Subsequently, HfO_2_ NPs were introduced and thoroughly sonicated to achieve a consistent dispersion. The resulting solution was then uniformly poured into a glass dish and dried at 60 °C to form the TPU-HfO_2_ composite film.

To assess the mechanical properties, tensile tests were conducted on the TPU-HfO_2_ composite films. Young’s moduli and elongation at break were determined using a Universal Testing Machine (Model QC-505M2F, Cometech, Taichung, Taiwan). The dielectric properties were evaluated using an Impedance Analyzer at room temperature, operating at a frequency of 100 kHz. The cross-sectional area’s morphological structure of the TPU-HfO_2_ composite film was observed through SEM. Mechanical stability was evaluated by assessing Young’s modulus and elongation at break using the same Universal Testing Machine.

The interaction between HfO_2_ NPs and TPU was analyzed using FT-IR (Nicolet iSTM50 spectrometer, Thermo Fisher Scientific). To assess the chemical stability of the TPU-HfO_2_ composite film, the composite films were subjected to a 35% strain for 21 days with FTIR spectra collected before and after the experiment. For examining changes in HfO_2_ NP distances under varying mechanical strain, AFM (XE-70, Park Systems, Suwon, Korea) was employed. A composite film measuring 20 × 5 mm was subjected to linear stretching at strain levels of 10%, 20%, 30%, 40%, and 50%, and the average particle distances were calculated using Gwyddion v2.63. The impact of linear strain on the electrical impedance of the TPU-HfO_2_ composite film was also investigated. During similar stretches, electrical impedance was continuously recorded over 250 s, demonstrating the measurement stability and consistency.

### *In Vitro* Tumor Volume Measurements

Simulated tumor models with varying volumes (25, 50, 75, 100, 200, 400, 500, 700, 1000, and 1200 mm) were created using a 3D printer (Sonic Mini 4K, Phrozen Technology, Taipei, Taiwan). These models were shaped like hemispheres. The DE strain sensor was the TPU-HfO_2_ composite film (diameter 20 mm), featuring two flexible carbon-tape electrodes (length 30 mm, width 3 mm, 3 mm apart). It was affixed onto a plastic ring, matching the simulated tumor’s diameter. This plastic ring consisted of interlocking inner and outer rings, which were also produced by using the same printer.

The stretching process was initiated by firmly pressing the plastic ring downward onto the simulated tumor model, causing the composite film to deform. Using an ELITE EIS data recorder (Wisetop Technology Co. Ltd., Hsinchu, Taiwan), we recorded the electrical impedances of both the unstretched (*Z*_0_) and stretched (*Z*) composite film were recorded. These impedances were then employed to calculate the relative impedance [(*Z*_0_ – *Z*)/*Z*_0_], providing the basis for generating the calibration curve against the simulated tumor volume.

The ELITE EIS recorder facilitated wireless data recording and transmission, utilizing the Wi-Fi networking standards IEEE 802.11b/g/n and Bluetooth 5.1. For recording electrical impedance, an AC voltage of 100 mV at a frequency of 20 kHz was employed. Throughout the study, carbon-tape electrodes were used as conductors.

To assess the accuracy and sensitivity of the DE strain sensor toward factors like size, shape, or individual variations, simulated tumor volumes (50, 100, 500, 1200 mm^3^) were transformed into four distinctive hemispheric configurations: one lobe (shape 1), two lobes (shape 2), three lobes (shape 3), and four lobes (shape 4). Across these various sizes and shapes, the DE strain sensor recorded electrical impedance readings that were measured by three different individuals. The acquired data were then translated into the corresponding tumor volumes using the established calibration curve. For comparison, the error percentage calculated by the DE sensor was compared with the error percentage derived from measurements taken with a caliper.

### Animal Study

Balb/c and Balb/c nude (nu/nu) mice (female, 6 weeks old) were procured from BioLASCO (Taipei, Taiwan). The experiments and handling of the mice adhered to the guidelines outlined in the “Guide for the Care and Use of Laboratory Animals”, created by the Institute of Laboratory Animal Resources, National Research Council, and published by the National Academy Press in 2011. The animal-related procedures were granted approval by the Institutional Animal Care and Use Committee of National Tsing Hua University (Approval Number 110005).

### Dose-Dependent Treatment Effectiveness of HfO_2_+US

After the inoculation of the cancer cells (1 × 10^6^ CT26 cells) subcutaneously into Balb/c mice, their tumor growth was closely monitored. When the tumor size reached 100 mm^3^, determined by measuring with a caliper, the mice were split into four groups: an untreated control group, a group receiving a single dose on day 0, a group receiving two doses on days 0 and 1, and a group receiving three doses on days 0, 1, and 2. Each treatment dose, administered through intratumoral injection into the tumor, consisted of 200 μg/mL of HfO_2_ NPs (0.1 mL) suspended in saline. This treatment was accompanied by US irradiation at an intensity of 1.0 W/cm^2^ for 10 min.

The mice’s body weights and tumor sizes, calculated using the formula length × width × height × π/6,^33^ were recorded every other day throughout the study. When the tumors in the untreated group reached a volume of 1200 mm^3^, the experiment was concluded. At this point, the mice were humanely euthanized and the tumors were collected and weighed using an electronic balance.

### *In Vivo* Tumor Volume Monitoring and Sonodynamic Therapy

After inoculation of the cancer cells (1 × 10^6^ CT26 cells) into Balb/c nude (nu/nu) mice, the progression of tumor growth was closely monitored using both the DE strain sensor and caliper techniques. To place the DE stain sensor on the inoculated tumor and record the electrical impedance, the mouse was put to sleep under isoflurane gas. The DE strain sensor with a diameter of 20 mm was positioned at the site of tumor inoculation on the mouse skin. A hole with a diameter of 17 mm was created in the Tegaderm film (3M, Maplewood, MN, USA) to affix it to the DE sensor around its outer edge, leaving a majority region unobstructed for its elastic stretching due to tumor growth. The silver cables from the ELITE EIS recorder were connected to the DE sensor for recording the electrical impedance.

To account for the potential influence of the surrounding tissue on the impedance signal, the DE sensor was placed on a tumor-free tissue, and its electrical impedance was recorded as a background signal. The impedance signal specific to the tumor was subsequently calculated by subtracting this background signal.^61^ This approach guarantees the effective mitigation of any interference stemming from the surrounding tissue, thereby upholding the accuracy and reliability of our measurements throughout the monitoring process.

The measurement recorded on day 0 served as the *Z*_0_, and the subsequent measurements were used as *Z* to calculate the tumor volume by using the calibration curve. The change in the tumor volume was displayed on the smartphone app wirelessly. To record the electrical impedance regularly, the mouse was exposed to isoflurane gas, and the measurements were recorded for 30 s everyday over the duration of the study. Concurrently, the change in tumor volume was also measured by a caliper regularly to compare the error % in the measurements against the DE sensor.

Once the tumor size reached 100 mm^3^, as determined by the DE strain sensor, the mice were divided into four groups. These groups were subsequently treated by intratumoral administration with one of the test formulations: untreated, US, HfO_2_ (200 μg/mL, 0.1 mL), or HfO_2_+US. The tumor volumes of the test mice, calculated using the formula length × width × height × π/6 with measurements from the caliper, were compared to the tumor volumes tracked using the DE sensor.

The mice were humanely euthanized once the tumors in the untreated group reached a volume of 1200 mm^3^. The tumors were collected and their volumes were assessed using a water displacement method.^62,63^ The tumor volumes measured by the caliper and DE strain sensor were then contrasted with the volumes determined through the water displacement technique. The discrepancy in measurements was calculated as a % error. The % error was determined by subtracting the volume measured using the caliper or DE sensor from the volume measured using the water displacement method and then dividing this difference by the volume measured through the water displacement method.

For histological analysis, the retrieved tumor tissues were fixed with 4% paraformaldehyde, embedded in paraffin wax (Tissue-Tek O.C.T. Compound, Sakura Finetek USA, Inc., Torrance, CA, USA), and then sectioned for H&E staining, Ki67 staining, or TUNEL assay. To evaluate its *in vivo* toxicity, the body weights were measured every day throughout the study and the vital organs of each studied group were retrieved at the end points, fixed in 4% paraformaldehyde, embedded in paraffin blocks, and stained with H&E for histopathological examination. To assess their potential liver and kidney toxicities, mouse sera were collected and the levels of AST, ALT, and BUN therein were measured.

### Statistical Analysis

All quantitative data are presented as the mean ± standard deviation. The two-tailed Student *t*-test was used to compare pairs of groups. A *P* value less than 0.05 was figured to be statistically significant.

## Supporting Information Available

Dimensions of HfO_2_ NPs determined through SEM analysis and subsequently analyzed using ImageJ software. Zeta potential value of HfO_2_ NPs measured using dynamic light scattering (DLS). Cell viabilities of NIH/3T3 cells following exposure to TPU-HfO_2_ composite film. FTIR spectra of TPU-HfO_2_ composite film both prior to and after subjecting it to a 35% strain for a period of 21 days. Dose-dependent treatment effectiveness of HfO_2_+US. Histopathological photomicrographs of tissue sections of major organs that were harvested from healthy and treated tumor-bearing mice. (PDF)
Video S1: Experimental setup for real-time evaluation of the status of tumor progression using the developed TPU-HfO_2_ DE strain sensor. (MP4)
Video S2: Monitoring tumor progression in an untreated mouse in real-time using a smartphone. (MP4)
Video S3: Real-time monitoring of tumor progression in a mouse treated with HfO_2_+US using a smartphone. (MP4)
