# Supplementary material for: Harnessing HfO2 Nanoparticles for Wearable Tumor Monitoring and Sonodynamic Therapy in Advancing Cancer Care
Source: ACS Nano. 2024 Jan 10;18(3):2485–99. doi: 10.1021/acsnano.3c11346 (PMC10811684; doi:10.1021/acsnano.3c11346)
Supplement: Supplementary file 1 — nn3c11346_si_001.pdf [file nn3c11346_si_001.pdf]

Supporting Information

Harnessing HfO_2 Nanoparticles for Wearable Tumor Monitoring and Sonodynamic Therapy in Advancing Cancer Care

Putry Yosefa Siboro[1,†], Amit Kumar Sharma[1,†], Pei-Jhun Lai[1], Jayachandran Jayakumar[1], Fwu-Long Mi[2], Hsin-Lung Chen[1], Yen Chang[3,], and Hsing-Wen Sung[1,*]*

[1] Department of Chemical Engineering, National Tsing Hua University, Hsinchu 30013, Taiwan (ROC)

[2] Department of Biochemistry and Molecular Cell Biology, School of Medicine, College of Medicine, Taipei Medical University, Taipei 23142, Taiwan (ROC)

[3] Taipei Tzu Chi Hospital, Buddhist Tzu Chi Medical Foundation and School of Medicine, Tzu Chi University, Hualien 97004, Taiwan (ROC)

KEYWORDS: tumor progression monitoring, hafnium oxide nanoparticle, wearable strain sensor, cancer care, telemedicine

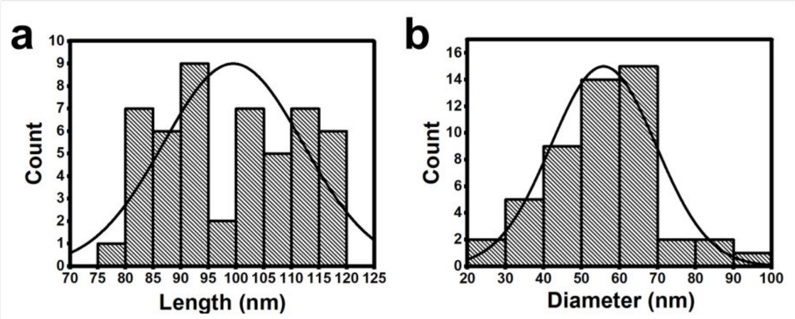

Figure S1. Dimensions of HfO_2 NPs, including their (a) length and (b) diameter, determined through SEM analysis and subsequently analyzed using ImageJ software.

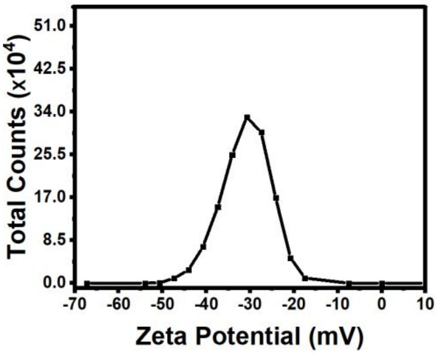

Figure S2. Zeta potential value of HfO_2 NPs measured using dynamic light scattering (DLS).

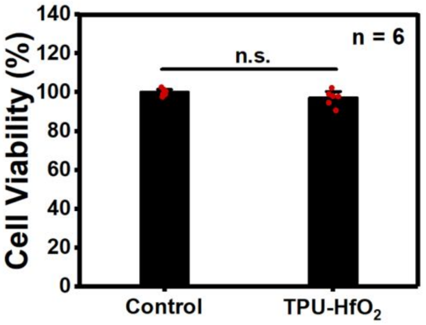

Figure S3. Cell viabilities of NIH/3T3 cells following exposure to TPU-HfO_2 composite film.

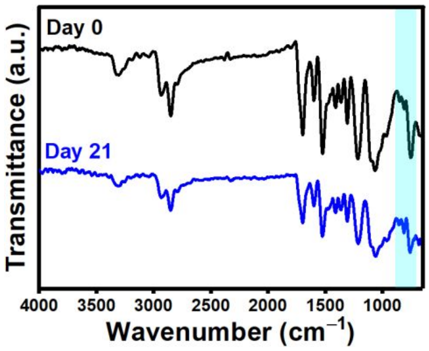

Figure S4. FTIR spectra of TPU-HfO_2 composite film both prior to and after subjecting it to a 35% strain for a period of 21 days.

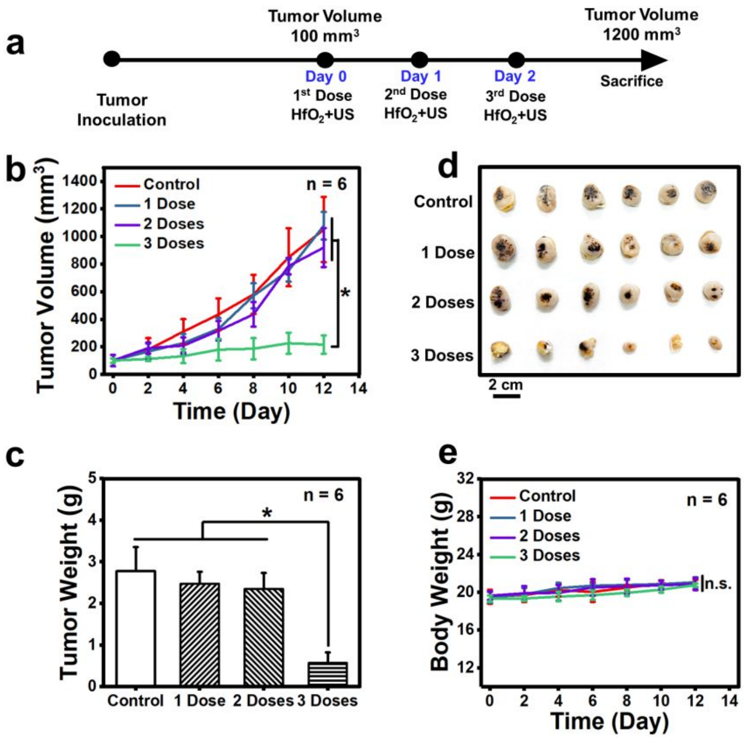

Figure S5. Dose-dependent treatment effectiveness of HfO_2+US. (a) Schematic time course of establishment of tumor model and subsequent treatment regimen. (b) Changes in tumor volumes, (c) harvested tumor weights, (d) photograph of harvested tumors, and (e) changes in body weights of test mice that had received different doses of HfO_2+US. *: statistically significant ($P < 0.05$); n.s.: not statistically significant. US parameters: 1.0 W/cm^2, 3.0 MHz, 10 min, and 50% duty cycle.

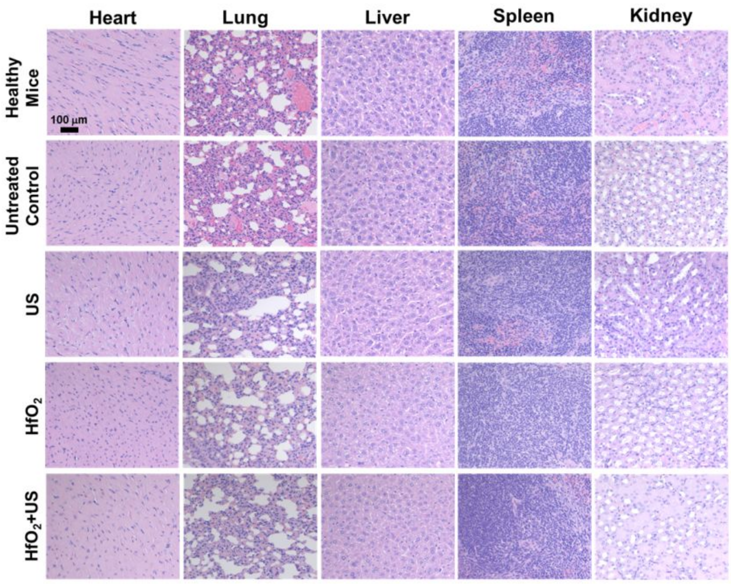

Figure S6. Histopathological photomicrographs of tissue sections of major organs that were harvested from healthy and treated tumor-bearing mice.

Video S1. Experimental setup for real-time evaluation of the status of tumor progression using the developed TPU-HfO_2 DE strain sensor.

Video S2. Monitoring tumor progression in an untreated mouse in real-time using a smartphone.

Video S3. Real-time monitoring of tumor progression in a mouse treated with HfO_2+US using a smartphone.
